# Effects of electron‐beam irradiation on inoculated *Listeria innocua,* microbiological and physicochemical quality of fresh noodles during refrigerated storage

**DOI:** 10.1002/fsn3.1277

**Published:** 2019-12-06

**Authors:** Feifei Shi, Hongwei Zhao, Hongbo Song, Weiling Guo, Li Wang, Xiaorui Cui, Weidong Zhang, Shurong Li

**Affiliations:** ^1^ Department of Food and Biological Engineering Beijing Vocational College of Agriculture Beijing China; ^2^ College of Food Science Fujian Agriculture and Forestry University Fuzhou China; ^3^ Qingdao University of Science and Technology Qingdao China; ^4^ Shandong Provincial Key Laboratory of Biochemical Engineering Qingdao China; ^5^ China Institute of Atomic Energy Beijing China

**Keywords:** Electron beam, Fresh noodles, *Listeria innocua*, Microbiota, Quality

## Abstract

As a nonthermal sterilization technology, electron‐beam irradiation (EBI) has attracted great interests for microbial inactivation in food preservation. In this study, the inactivation of inoculated *Listeria innocua*, natural microbiota, and quality of fresh noodles treated by EBI during refrigerated storage were evaluated. Results showed that the initial *L. innocua* population (6.38 log CFU/g) was significantly reduced to an undetectable level by treatment with 3.0 kGy EBI. Moreover, treatment with 3 kGy EBI significantly reduced the initial total bacteria counts and fungal counts (mold and yeast) from 5.66 and 3.15 log CFU/g to 2.90 and 2.11 log CFU/g, respectively. However, along with the storage process, the inoculated *L. innocua* and natural microbiota were recovered resulting in the increased populations of the spoilage microorganisms. Increasing the dose of EBI to 4.0 kGy or 5.0 kGy, the *L. innocua* population was inhibited to the undetectable level and the microbiological quality of the fresh noodles was kept in the acceptable level during the 28 day storage. In addition, changes of the physicochemical indicators including pH value, color, cooking characteristics, texture, and sensory of fresh noodles treated with EBI were delayed during storage. These results reveal that EBI treatment can improve the microbiological safety and shelf life of fresh noodles without impairing quality.

## Graphical abstract

As a non‐thermal sterilization technology, electron beam irradiation (EBI) has attracted great interests for microbial inactivation in food preservation. In this study, the inactivation of inoculated *Listeria innocua*, natural microbiota and quality of fresh noodles treated by electron beam irradiation (EBI) during refrigerated storage were evaluated. Results revealed that EBI treatment could be used to improve the microbiological safety and shelf life of fresh noodles without impairing quality.

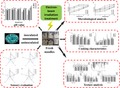

## 1 INTRODUCTION

Noodles are regarded as a traditional human diet, which have been consumed for thousands of years in many Asian countries, such as China, Korea, Japan, and Thailand, due to its convenience, nutritional quality, and palatability (Heo, Lee, Shim, Yoo, & Lee, 2013; Li, Zhu, et al., 2012a). In particular, the fresh noodles, which possess unique flavor and taste, are attracting more and more consumer preferences (Hou, 2001). However, due to the high water content and rich nutrients, the fresh noodles could be easily contaminated by foodborne pathogens and spoilage microorganisms (Li, Zhang, et al., 2012b), resulting in a quite short shelf life even if they are stored under refrigeration conditions (Ghaffar, Abdulamir, Bakar, Karim, & Saari, 2009) in which psychrophilic microorganisms could survive and grow on the refrigerated foods (Riebroy et al., 2007). Previous research has shown that the very common pathogenic psychrophilic bacteria, *Listeria monocytogenes*, could proliferate on the fresh noodles at refrigerated temperature and causes listeriosis disease for human (Mahmoud, 2012). Therefore, keeping the higher quality and extending shelf life of fresh noodles are still big challenges in the current, which will result in the huge waste in food industry and potential food poisonings. At present, the extensively used sterilization methods of fresh noodles are adding various food preservatives, such as flaxseed flour, glycinin basic polypeptide, and so on (Hou, Li, Wang, Sun, & Mo, 2017; Xu, Hall, Wolf‐Hall, & Manthey, 2008). Nevertheless, preservatives may result in changing the flavor of fresh noodle and disrupting the nutritional value of fresh noodle as well as limited antibacterial activity. Thus, it is important to reinforce researches into a novel sterilization technology that could effectively prolong the shelf life of fresh noodle and maintain their flavor and taste.

With the development of nonthermal technologies, ionizing radiation has been regarded as an effective bactericidal technology that aims to achieve both microbiological safety and minimum loss of food nutrition and quality characteristics. The most common sources of ionizing radiation are γ‐irradiation, X‐rays, and electron‐beam (EBI) (Farkas & Mohácsi‐Farkas, 2011). Compared with other irradiation methods, EBI, derived from high‐energy electron beams, has been used as a clean and environmentally friendly technology for industrial food processing and is regarded as a potential novel nonthermal sterilization with a greater power utilization rate and a lower operation cost (Wei et al., 2014). Most of the spoilage or pathogenic microorganisms could be killed by the treatment of EBI, caused by the destructive effects of the generated free radicals on the structures of DNA, proteins, and other biomacromolecules (Acheson & Steele, 2001). EBI has been used in many foods for sterilization such as mushrooms, blueberries, cantaloupe, spinach leaves, and so on (Ehlermann, 2005; Fernandes, Antonio, Oliveira, Martins, & Ferreira, 2012; Gomes et al., 2010; Moreno, Castellperez, Gomes, Da, & Moreira, 2007; Wei et al., 2014). Different foods may have different sensitivities to irradiation due to their different compositions. Kong et al. (2014) reported that EBI effectively inactivated *Escherichia coli* inoculated on fresh blueberries and extended the shelf life of fresh blueberries without changing nutritional quality at doses ≤ 3 kGy. Feng, Jo, Nam, and Ahn (2019) found that EBI < 4.5 kGy was effective doses for the vacuum‐packaged raw ground beef without influencing its physicochemical characteristics.

As far as we know, few studies have focused on the application of EBI to the preservation of noodle products. Therefore, in this study we explored the sterilization effects of EBI on the inoculated *L. innocua* and natural microbiota in fresh noodles, as well as physicochemical quality including pH value, color, cooking characteristics, texture, and sensory during refrigerated storage. This study will provide better insights into the potential applications of EBI in the future.

## 2 MATERIALS AND METHODS

### 2.1 Fresh noodles preparation

The formula of fresh noodles consisted of 100 g wheat flour and 34 ml distilled water and was evenly mixed to prepare the dough. After a 20‐min rest, the prepared dough was passed through an automatic noodle machine (Model JYS‐N6, Joyoung, Co., Ltd.,) to make the noodle strands. The prepared fresh noodles were weighed and subpacked in PE bags every 25 g. Two independent groups of fresh noodles were set: one was inoculated with *L. innocua* before EBI treatment and the other was directly irradiated with EBI for microbiological and physicochemical quality analysis.

### 2.2 Bacterial culture and inoculation

*Listeria innocua* 1.2990, a nonpathogenic species purchased from the China General Microbiological Culture Collection Center (CGMCC), was selected as a surrogate of *L. monocytogenes* as the two strains showed similar radiation resistance (Kamat & Nair, 1996). Cultures were made by inoculating one single colony from a Trypticase Soy‐Yeast Extract Agar (TSA‐YE, Beijing Land Bridge Technology Co., Ltd.,) plate in 10 ml of Trypticase Soy‐Yeast Extract Broth (TSB‐YE) and cultivating in a shaker at 37°C and 200 r/min for 24 hr. The cultures were reinoculated (3% v/v) into TSB‐YE and incubated at 37°C for 16 hr under constant shaking (200 r/min). The cells were harvested by centrifugation (8,000 × g, 10 min, 4°C) and washed three times with 0.85% sterile saline. The fresh noodles were irradiated at 5.0 kGy before inoculation to remove pre‐existing microorganisms (inoculation group). The prepared inoculum solution (250 μL) was evenly spread onto the surfaces of the fresh noodles (25 g) using an injector with an initial inoculum of approximately 6–7 log CFU/g. The inoculated fresh noodles were then air‐dried in the biosafety cabinet for 30 min to allow bacterial attachment before EBI treatments.

### 2.3 EBI treatment

EBI treatment processing of fresh noodles was carried out at room temperature in China Institute of Atomic Energy Beijing, China, using an electron beam accelerator (DZ‐12/4) with the following parameters: energy of 10 MeV, power of 4 kW, and scan width of 50 cm. The inoculated samples were irradiated at doses of 0.5, 1.0, 1.5, 2.0, 2.5, 3.0, 4.0, and 5.0 kGy, and noninoculated samples were irradiated at doses of 1.0, 2.0, 3.0, 4.0, and 5.0 kGy. All the experiments were run for 3 times. The nonirradiated samples were used as controls. All samples were stored at 4°C, and sampling was taken at a day 0, day 7, day 14, day 21, and day 28 for the analysis of total microbial counts and quality characteristics.

### 2.4 Microbiological analysis

Twenty‐five gram of fresh noodles were homogenized with 225 ml sterile saline (0.85% NaCl) using a sterile homogenizer (Scientz‐09, Ningbo Scientz Biotechnology Co., Ltd.,) for 2 min. Gradient dilutions were prepared with sterile saline for plate counts of inoculated *L. innocua* on PALCAM with additives at 37°C for 24 hr. For the total bacterial counts (TBC), 1 ml of the appropriate dilutions was evenly added to plate count agar and incubated at 37°C for 24 hr; for the mold and yeasts counts (MYC), rose bengal medium was used and incubated at 28°C for 5 days. Viable counts were expressed as log CFU/g.

### 2.5 pH value

Ten grams of fresh noodles were mixed with 90 ml of distilled water in a stomacher for 2 min. Then, 10 ml of the mixture was homogenized and the pH were measured with a pH meter (PHS‐3C) (Lhomme et al., 2016).

### 2.6 Measurement of color

Color of the noodles was determined by a color meter (WSC‐S, Shanghai precision & scientific instrument Co. Ltd.,). The color values L* and b* were measured after calibration of the colorimeter by using white and black standards (Khouryieh, Herald, & Aramouni, 2010).

### 2.7 Cooking characteristics

Cooking characteristics were performed based on previous studies with some modifications (Fan, Ma, Wang, & Zheng, 2016; Li, Luo, et al., 2012c; Supawadee & Prisana, 2010). Ten grams of noodles was placed in 500 ml of boiling water and cooked to the optimum cooking time. Cooked noodles were washed with distilled water and drained for 5 min before weighting. Water absorption was calculated based on formula (1). After the noodles cooking, the water was collected in a 500 ml volumetric flask to make a constant volume, and then, 100 ml of the solution was dried to a constant weight with a laboratory oven at 105°C. The residue was weighed and cooking loss was calculated by formula (2).

(1) $$\text{Water absorption}\;=\;\frac{\text{Weight of cooked noodels (g) - Weight of uncooked noodles (g)}}{\text{Weight of cooked noodels (g)}}$$

(2) $$\text{Cooking loss}\;=\;\frac{\text{Weight of dried residue (g)}}{\text{Weight of uncooked noodles (g)}}$$

### 2.8 Texture analysis

The cooked noodles were prepared, and textural quality including hardness, adhesiveness, gumminess, chewiness, and springiness were determined by a Texture Analyzer (Model TAplus). The testing parameter of the TPA test was as follows: test speed of 8 mm/s, target value of 1 mm, recovery time of 1 s, and trigger point of 5 g.

### 2.9 Sensory evaluation

The sensory characteristics of nonirradiated and irradiated stored samples were evaluated by ten untrained panelists. The following sensory attributes were evaluated: surface condition, odor, palatability, and overall acceptability. As fresh noodles deteriorated at the end of storage, sensory evaluation was stopped when the microbial counts exceeded the upper limit (10^6^ CFU/g) (Lainez, Vergara, & Bárcenas, 2008). For each parameter, nine‐point hedonic scale was used, ranging from 1 (dislike extremely) to 9 (like extremely). A score of 5 or below was regarded as the end of shelf life.

### 2.10 Statistical analysis

All data were subjected to one‐way ANOVA performed with SPSS 17.0 software (IBM Corp., Armonk) and presented as the mean ± standard deviation. Statistically significant differences were considered at *p* < .05.

## 3 RESULTS AND DISCUSSION

### 3.1 Effect of EBI on the survival of inoculated *L. innocua*

The inactivation effects of different EBI doses on inoculated *L. innocua* of fresh noodles during refrigerated storage were evaluated, and the results were shown in Table 1. A clear dose‐related decrease in total counts of inoculated *L. innocua* of fresh noodles was observed. An approximate 1.39 log CFU/g reduction was obtained by 0.5 kGy EBI treatment compared with the control group, which is in agreement with previous studies that effective inactivation could be obtained at low irradiation doses (Chun, Kim, Lee, Yu, & Song, 2010; Nam et al., 2006). The initial amount of *L. innocua* inoculated on fresh noodles was 6.38 log CFU/g, which was significantly reduced to the undetectable level in the sample treated with EBI at 3.0 kGy within 7 days of storage. However, the *L. innocua* recovered from the EBI‐treated fresh noodles and the total cell counts increased up to 3.15 log CFU/g after 28 days of refrigerated storage. Increasing the dose of EBI to 4.0 kGy or 5.0 kGy, the population of *L. innocua* inoculated on fresh noodles could be kept at the undetectable level during the whole period of storage. These results were in agreement with those obtained by Mahmoud (2012) who found that *L. monocytogenes* was able to recover and grow in smoked salmon treated with irradiation at less than 2.0 kGy during storage at refrigerated temperature, which could result in a high risk of listeriosis disease (Sudha, Lammerding, & Griffiths, 2000). Low dose of EBI treatment could cause temporary dormancy of the microbial cells with the direct and/or indirect effects (Lopatina, Zadereev, Oskina, & Petrichenkov, 2018; Selim, Saha, & Mukherjea, 2017). Therefore, the surviving cells could trigger the DNA repair systems for self‐healing (Repar et al., 2017). A previous research showed that in the case of low‐dose irradiation, the detected DNA damages were completely repairable, whereas the high‐dose irradiation demonstrated a lower level of reparability (Babayan et al., 2017). In order to control the spoilage microorganisms in safety level, relatively high‐dose irradiation is required. Our results showed that EBI treatment dose at 4 kGy or above could be used to control the contamination caused by *Listeria* in the storage of fresh noodles*.*

**Table 1:** Changes in populations of *L. innocua* inoculated on fresh noodles irradiated by different doses during refrigerated storage

Irradiation dose/kGy	Total counts of *L. innocua*/log CFU/g				
	0 d	7 d	14 d	21 d	28 d
0.0	6.38 ± 0.13	6.51 ± 0.13	7.12 ± 0.10	8.14 ± 0.18	8.17 ± 0.21
0.5	4.99 ± 0.36	5.23 ± 0.18	5.43 ± 0.16	6.20 ± 0.23	7.10 ± 0.13
1.0	3.96 ± 0.32	4.10 ± 0.16	4.90 ± 0.09	5.34 ± 0.07	6.43 ± 0.19
1.5	2.89 ± 0.11	2.93 ± 0.03	3.40 ± 0.18	3.75 ± 0.18	5.19 ± 0.17
2.0	1.92 ± 0.08	1.94 ± 0.12	3.04 ± 0.08	3.14 ± 0.14	4.80 ± 0.04
2.5	1.19 ± 0.13	1.20 ± 0.14	1.81 ± 0.09	2.91 ± 0.13	4.44 ± 0.13
3.0	ND	1.28 ± 0.27	1.67 ± 0.15	2.37 ± 0.35	3.15 ± 0.11
4.0	ND	ND	ND	ND	ND
5.0	ND	ND	ND	ND	ND

Abbreviation: ND means “not detected.”

### 3.2 Effect of EBI on the microbiological quality of fresh noodles

**Figure 1:**
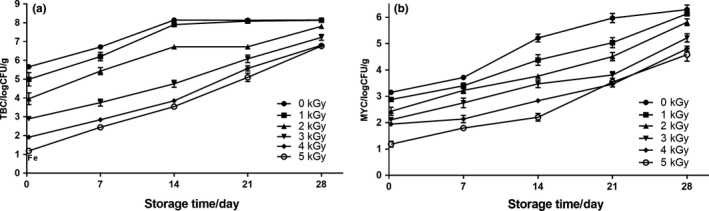
Effects of EBI on the microbial populations of fresh noodles during refrigerated storage. A, TBC (total bacterial counts); B, MYC (mold and yeast counts)

Microbiological quality determined the shelf life of fresh noodles. Therefore, in this study, the effects of different irradiation doses on the natural microbiota population of the fresh noodles during refrigerated storage were evaluated. As shown in Fig. 1, a dose‐dependent reduction was observed in TBC and MYC. The initial TBC in the fresh noodle sample was 5.66 log CFU/g, and it could be increased up to about 8 log CFU/g within 14 days if the noodles were not treated with any EBI treatment, indicating the end of the shelf life (Rahman, Jin, & Oh, 2011). Initial TBC of the noodles treated with EBI at 1.0 kGy, 2.0 kGy, 3.0 kGy, 4.0 kGy, and 5.0 kGy was significantly decreased by 0.66, 1.69, 2.76, 3.73, and 4.46 log CFU/g compared to the control group (*p* < .05), respectively (Fig. 1a). Although the TBC of fresh noodles significantly increased along with the extension of storage time in all samples (*p* < .05), EBI could obviously slow the growth of the spoilage microorganisms. Moreover, TBC could be controlled under the safe level of 7 log CFU/g in fresh noodles treated with EBI at 3.0 kGy or above during the 28 day storage.

Similar trends were also found in the change of the MYC of the noodles. As shown in Fig1b, the levels of MYC were reached 5.22 log CFU/g in the nontreatment group within 14 days of refrigerated storage, which could be regarded as spoiled food (Rahman, Wang, & Oh, 2013). The MYC of fresh noodles in the 1.0–5.0 kGy irradiated groups were significantly decreased compared with the control group, especially the dose of 3.0 kGy and above which could control the MYC of fresh noodles under 5 log CFU/g and extend the shelf life to 28 days.

Higher water activity and neutral pH of fresh noodles could boost the proliferation of spoilage microorganisms resulting in a relatively short shelf life, which cause heavy burden on the cost of food distribution and safety of consumption (Huang, Huang, Huang, & Chen, 2007). Our data revealed that the shelf life of fresh noodle treated with EBI at 3.0 kGy and above could be extended to 28 days, which was in good agreement with previous results that irradiation dose of 3.0 kGy produced immediate reduction of 4 log units of the total aerobic plate counts in ground beef (Javanmard, Rokni, Bokaie, & Shahhosseini, 2006). Considering the contamination of the foodborne pathogens, we concluded that 4.0 kGy or 5.0 kGy irradiation treatment could be suitable for the sterilization of the fresh noodles before preservation.

### 3.3 Effect of EBI on the physicochemical quality of fresh noodles

#### 3.3.1 Effect on pH value

**Figure 2:**
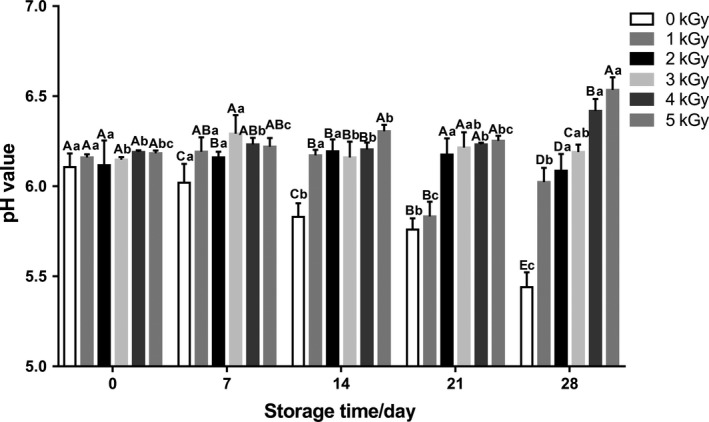
Effects of EBI on the pH values of fresh noodles during refrigerated storage. Data are presented as the means ± standard deviation. Means within same storage time at different EBI dose with different capital letters are significantly different (*p* < .05); means for same EBI dose at different storage time with different small case letters are significantly different (*p* < .05)

The pH value is considered to be an important food quality index during storage process (Cheon, Seo, Chung, & Chun, 2016; Pinela et al., 2016). The effects of EBI on the pH values of fresh noodles during refrigerated storage were studied, and the results were shown in Fig. 2. There were no significant differences in initial pH value between the irradiated and nonirradiated samples, which is in agreement with the previous research that irradiation treatment had no significant effect on the pH of beef sausages (Badr & Mahmoud, 2011). Along with the storage, the pH value of the control significantly dropped from 6.11 to 5.44 within 28 days of storage. However, the pH of EBI‐treated samples with moderate high dose (>2.0 kGy) remained higher level. The decrease of pH values in foods could adversely affect the sensory quality, resulting from the spoilage microorganisms’ metabolism, especially in food rich in carbohydrates, which can be utilized by microorganisms to produce acids (Cheon et al., 2016). Our results showed that EBI treatment could slow down the decrease of pH values to keep the fresh noodles in high quality during the storage*.*

#### 3.3.2 Effect on color

The color is a critical factor to consumer acceptance and the success of food products (Fu, 2008). Effects of EBI on the color of fresh noodles during refrigerated storage were studied. Two color parameters (L* and b*) were measured, in which L* and b* reflect the brightness and yellowness of the noodles, respectively. As shown in Table 2, among all the samples, the L* values of the fresh noodles steadily decreased and b* values increased during 28 day of refrigerated storage, which can be attributed to the enzymatic browning of fresh noodles probably caused by polyphenol oxidase during storage time (Asenstorfer, Appelbee, & Mares, 2010; Cortés, Esteve, & Frígola, 2008). However, treatment with EBI could significantly reduce the changes of the L* and b* values compared with the control group, especially in 5 kGy treatment group, suggesting that higher dose of EBI treatment could maintain the high quality of color property in fresh noodle during storage process, which is in agreement with previous studies (Siriporn et al., 2007; Sirisoontaralak & Noomhorm, 2007). EBI is capable of destroying the protein structures to reduce the activity of polyphenol oxidase (Wang et al., 2017). Moreover, previous study has shown that antioxidants could be formed by γ‐irradiation through the formation of Maillard reaction products (MRPs) from glucose and lysine/glycine (Chawla, Chander, & Sharma, 2007). The damaged polyphenol oxidase and produced antioxidants could alleviate the deterioration of color property during fresh noodle storage.

**Table 2:** Effects of EBI on the color of fresh noodles during refrigerated storage

Color	Irradiation dose/kGy	Storage time/d				
		0	7	14	21	28
Brightness /L*	0	76.08 ± 3.15^Aa^	65.49 ± 2.22^Cb^	61.12 ± 2.57^Bc^	50.85 ± 3.09^Dd^	41.50 ± 10.76^De^
	1	76.10 ± 1.00^Aa^	67.14 ± 1.54^BCb^	62.49 ± 2.41^Bc^	54.60 ± 4.06^Dd^	43.18 ± 3.10^De^
	2	76.34 ± 1.16^Aa^	68.74 ± 1.78^Bb^	63.11 ± 1.69^Bb^	65.14 ± 1.79^Cc^	49.17 ± 2.59^Cd^
	3	76.24 ± 1.23^Aa^	68.26 ± 3.20^Ba^	64.40 ± 2.30^Bb^	60.16 ± 2.81^Cb^	52.74 ± 3.16^Bc^
	4	76.50 ± 1.58^Aa^	68.87 ± 2.80^Bb^	69.78 ± 4.09^Ab^	71.85 ± 7.22^Bb^	58.62 ± 2.23^Bc^
	5	76.88 ± 1.64^Aa^	74.61 ± 2.63^Aa^	71.82 ± 4.22^Aa^	76.11 ± 2.24^Aa^	68.19 ± 2.45^Ab^
Yellowness /b*	0	15.69 ± 1.31^Ab^	17.81 ± 0.65^Ab^	20.34 ± 1.16^Ab^	25.84 ± 1.44^Ab^	60.11 ± 4.52^Aa^
	1	15.63 ± 0.77^Ab^	16.91 ± 0.87^Ab^	20.12 ± 0.97^Ab^	22.08 ± 2.16^Bb^	50.89 ± 7.91^Ba^
	2	15.67 ± 0.87^Ac^	16.65 ± 1.10^Ac^	19.90 ± 0.82^Bb^	21.59 ± 0.63^Bb^	39.10 ± 4.63^Ca^
	3	15.50 ± 0.48^Ac^	16.57 ± 1.08^Bc^	18.72 ± 1.36^Bb^	20.60 ± 3.80^Bb^	29.40 ± 4.37^Da^
	4	15.38 ± 0.70^Ab^	15.69 ± 0.77^Bc^	15.94 ± 3.15^Cb^	17.33 ± 4.94^Cb^	22.04 ± 4.37^Ea^
	5	15.14 ± 0.61^Ab^	15.77 ± 2.24^Bb^	15.83 ± 1.27^Cb^	16.58 ± 0.95^Db^	18.31 ± 5.11^Fa^

Note

Means within same storage time at different EBI dose with different capital letters are significantly different (*p* < .05); means for same EBI dose at different storage time with different small case letters are significantly different (*p* < .05).

#### 3.3.3 Effect on cooking characteristics

**Figure 3:**
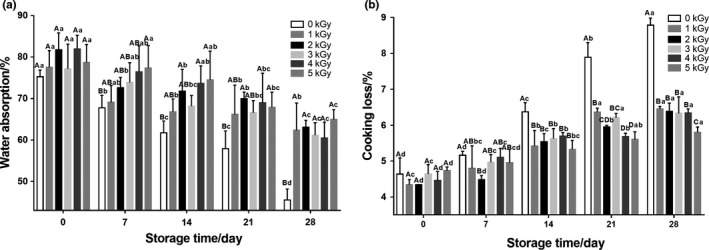
Effects of EBI on the cooking characteristics of fresh noodles during refrigerated storage. Data are presented as the means ± standard deviation. Means within same storage time at different EBI dose with different capital letters are significantly different (*p* < .05); means for same EBI dose at different storage time with different small case letters are significantly different (*p* < .05). A, water absorption; B, cooking loss

Two cooking quality indexes, water absorption and cooking losses of the fresh noodles, were studied, and the results were shown in the Fig. 3. Significant decreases in the water absorption were observed in all the noodle samples along with the extension of storage time (Fig. 3a). However, EBI treatment could delay the reduction trends with dose‐dependent effects. The noodles treated with EBI at 5.0 kGy showed a higher water absorption of 64.94% after 28 days storage compared to other samples. It could be ascribed to the degradation of starch chains, fiber, polysaccharide, and protein caused by EBI treatment. The resulted fragments of the biomacromolecule in EBI treatment groups contributed more water binding sites that could enhance the water holding capacity, as reported extensively in the previous study (Ashwar et al., 2014).

Cooking loss was an important parameter in the quality of fresh noodles, which was defined as the amount of solid substance lost into cooking water (Li, Luo, et al., 2012c). As shown in Fig. 3b, a significant increase of the cooking loss was observed during the 28 day storage of fresh noodles with or without EBI treatment. In addition, the initial cooking loss showed no significant differences among the samples, which indicated that EBI itself could not enhance the cooking loss of fresh noodles in a short time. Our results were in agreement with the previous study that low‐dose irradiation had no significant effects on cooking loss of pork loins (Mattison et al., 1986). Interestingly, the cooking loss in the EBI treatment groups was significantly lower than that of the control group at the stage of the storage (day 14, day 21, and day 28) (*p* < .05). It could be attributed to that EBI would promote protein aggregation to form a stronger 3‐D network entrapping starch granules and preventing them leaching into the cooking water in a slow speed (Zweifel, Handschin, Escher, & Conde‐Petit, 2003).

#### 3.3.4 Effect on texture

**Figure 4:**
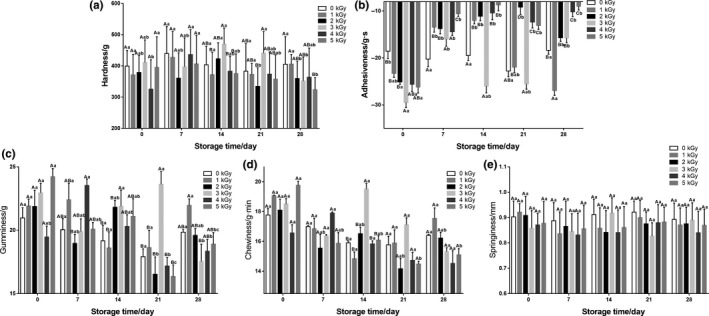
Effects of EBI on the texture attributes of fresh noodles during refrigerated storage. Data are presented as the means ± standard deviation. Means within same storage time at different EBI dose with different capital letters are significantly different (*p* < .05); means for same EBI dose at different storage time with different small case letters are significantly different (*p* < .05). A, hardness; B, adhesiveness; C, gumminess; D, chewiness; and E, springiness

The texture characteristics of cooked noodles are the focus of the consumers concern in choosing high‐quality noodle products (Khouryieh et al., 2010). As shown in Fig. 4, there were no significant differences (*p* > .05) in hardness, gumminess, springiness, and chewiness among the all experimental groups compared to the control at the initial of storage, which is in agreement with previous study (Velasco, Ordóñez, Cabeza, & Cambero, 2016). As the storage time extended, the texture indexes including hardness, adhesiveness, chewiness, and gumminess in the control group showed a significant increase, while these indexes in irradiated groups just decreased slightly with no obvious downward trends, which are consistent with the results obtained by Lee, Kang, and Kim (2017). However, no significant changes in springiness were observed during storage in irradiated groups and control group (*p* > .05). During the storage time, the moisture of fresh noodles migrated to the surface, resulting in an uneven water distribution and making it difficult for water to enter the center of the noodle. At the same time, the large amount of microbial growth and fermentation with acid production would have certain effects on the gluten network structure, resulting in the increase of hardness and chewiness (Li, Ma, Zhu, Guo, & Zhou, 2017).

#### 3.3.5 Effect on sensory evaluation

**Figure 5:**
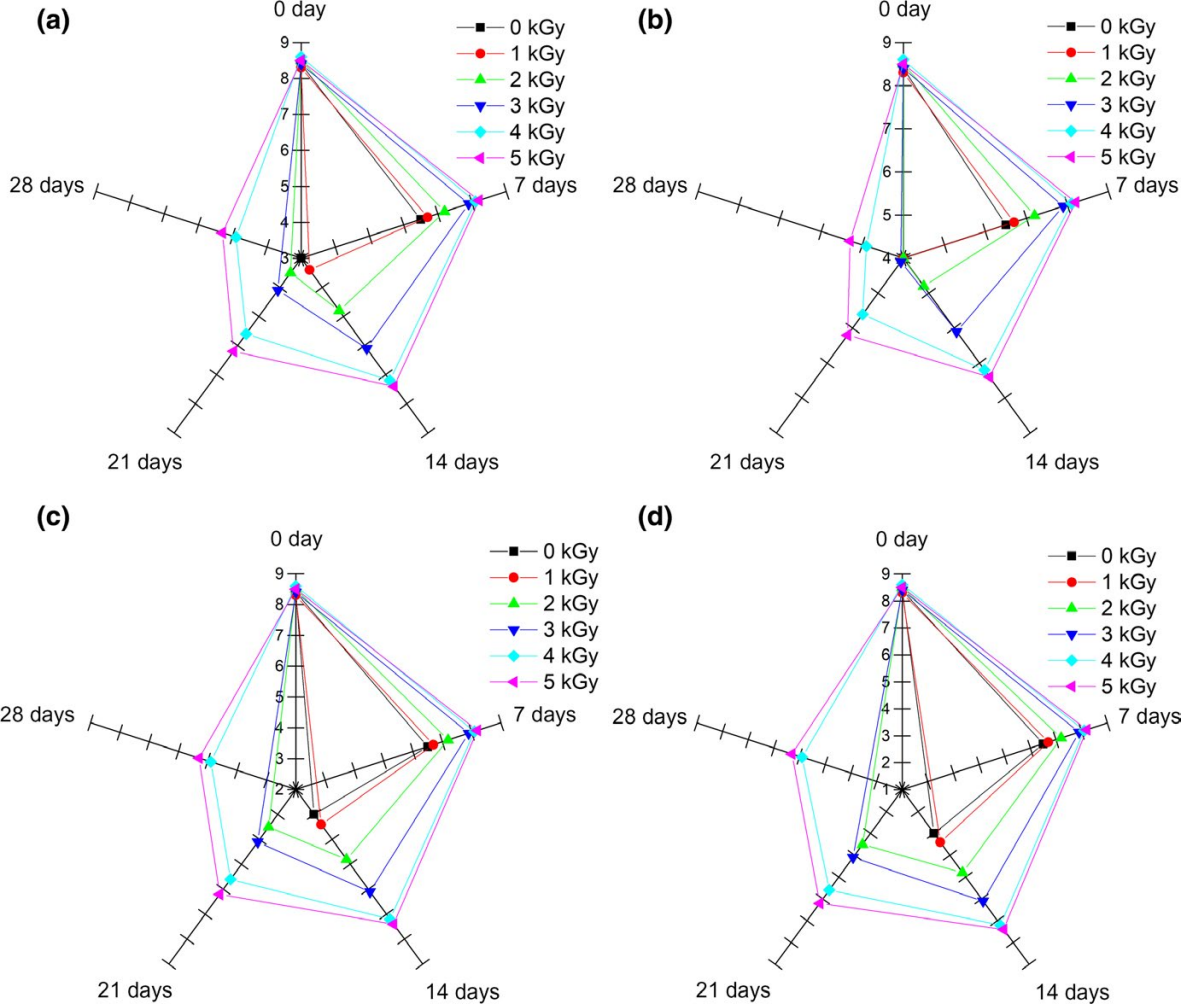
Effects of EBI on the sensory attributes of fresh noodles during refrigerated storage. A, surface condition; B, odor; C, palatability; and D, overall acceptability

Sensory quality of the noodles samples was evaluated by a sensory panel throughout the storage period. Noodles sensory attributes such as surface condition, odor, palatability, and overall acceptability are closely associated with the structural and textural properties of cooked noodles. Fig. 5 illustrated the sensory properties of fresh noodles exposed to different EBI treatment doses. EBI at the selected doses had no adverse effects on the sensory properties of the fresh noodles at the initial of storage (i.e. surface condition, odor, palatability, and overall acceptability) (*p* > .05). Considering the safety of the noodles, sensory evaluation would be stopped as the fresh noodles exceeded the shelf life. During refrigerated storage, all sensory attribute scores significantly decreased with increasing storage time. Based on the reported spoilage index that if the score of the overall acceptability was less than 5, the product would be regarded as putrid food (Lainez et al., 2008). Our data showed that the control samples had significantly lower sensory attribute scores than that of irradiated samples during storage time (*p* < .05). It could be attributed to high lipid oxidation and the metabolites of spoilage microorganisms (Maqsood & Benjakul, 2010). Samples irradiated at 5.0 kGy still showed acceptable sensory quality at 28 days, indicating that EBI treatment could maintain the quality during the noodle storage.

## 4 CONCLUSIONS

In conclusion, our study clearly indicated that EBI could decrease the populations of inoculated *Listeria innocua* and natural microflora as well as maintaining higher quality of fresh noodles during refrigeration storage. At the doses of 4.0 kGy or 5.0 kGy, the *L. innocua* population was inhibited to the undetectable level and the microbiological quality of the fresh noodles was kept in the acceptable level during the 28 day storage. In addition, changes of the physicochemical indicators including pH value, color, cooking characteristics, texture, and sensory of fresh noodles treated with EBI were delayed during storage. Higher dose of EBI (4.0 kGy or 5.0 kGy) could be suitable for the sterilization of the fresh noodles before preservation. Further works need to be focused on the mechanisms of sterilization and preservation, as well as the relationships between the dynamic changes of the natural microbiota and the quality of the fresh noodles during the storage.

## CONFLICT OF INTEREST

The authors declare that the research was conducted in the absence of any commercial or financial relationships that could be construed as a potential conflict of interest.

## ETHICAL STATEMENT

The study did not involve any human or animal testing.
